# Do True and False Intentions Differ in Level of Abstraction? A Test of Construal Level Theory in Deception Contexts

**DOI:** 10.3389/fpsyg.2017.02037

**Published:** 2017-11-21

**Authors:** Sofia Calderon, Erik Mac Giolla, Pär Anders Granhag, Karl Ask

**Affiliations:** ^1^Department of Psychology, University of Gothenburg, Gothenburg, Sweden; ^2^Norwegian Police University College, Oslo, Norway

**Keywords:** true and false intentions, mental representations, deception, Construal Level Theory, psychological distance

## Abstract

The aim was to examine how people mentally represent alleged future actions—their *true* and *false* intentions. In two experiments, participants were asked to either tell the truth (i.e., express true intentions) or lie (i.e., express false intentions) about performing future tasks. Drawing on Construal Level Theory, which proposes that psychologically distant events are more abstractly construed than proximal ones, it was predicted that liars would have more abstract mental representations of the future tasks than truth tellers, due to differences in hypotheticality (i.e., the likelihood of the future tasks occurring). Construal level was measured by a video segmentation task (Experiment 1, *N* = 125) and preference for abstract or concrete descriptions of tasks (Experiment 2, *N* = 59). Veracity had no effect on construal level. Speaking against our initial predictions, the data indicate that true and false intentions are construed at similar levels of abstraction. The results are discussed in the light of Construal Level Theory and the emerging psycho-legal research on true and false intentions.

## Introduction

All situations which are not directly experienced are cognitively represented in some form. Construal Level Theory (CLT) provides a framework for understanding how such representations are construed (Trope and Liberman, 2010). CLT proposes that peoples’ mental representations differ in systematic and predictable ways, from higher-level, abstract construals to lower-level, concrete construals. In the current study, we extend CLT to how we mentally represent truths and lies. Specifically, we examine the special case of *true* and *false* intentions. Since credibility judgments of verbal statements play a central role in many applied settings, it is crucial to understand the mental representations on which such statements are based. As will be explained below, theory and empirical research indicate that there should be differences in how true and false intentions are mentally represented. This can provide one potential route to aid in the task of discriminating between true and false intentions in real life legal situations.

### Construal Level Theory

Construal Level Theory (CLT) was developed to systematize and explain how we mentally represent events (or objects) that are not in the here and now, such as memories of the past and thoughts about the future (Trope and Liberman, 2010). In brief, the theory proposes that events can be more or less psychologically distant, and the more distant an event is perceived to be, the more abstractly it will be represented. A large number of empirical studies support the theory, by showing that increasing psychological distance leads to more abstract mental representations (for a recent meta-analysis, see Soderberg et al., 2014).

Four types of psychological distance have been proposed; temporal, spatial, social, and hypothetical. The specific form of psychological distance known as hypotheticality (likelihood) is of particular interest for the current study. According to CLT the perceived likelihood of an event influences how it is mentally represented. Specifically, unlikely events are claimed to be more psychologically distant than likely events. Accordingly, the mental representations of such unlikely events should be represented at a relatively higher, more abstract level than likely events. In a series of experiments, Wakslak et al. (2006) tested this claim by manipulating the likelihood of future events and measuring how this affected people’s mental construal of these events. In accordance with CLT they found that more unlikely events were represented at higher construal levels. For example, in one experiment, participants were told there was either a 95% or a 5% chance that they were going to perform a future task. Participants in the low likelihood condition segmented the task, which was depicted in a video clip, into fewer meaningful action units (Wakslak et al., 2006, Experiment 4). In other experiments in the abovementioned study, participants in low likelihood conditions showed a greater preference for abstract (than concrete) descriptions of a task (Experiment 3), and grouped objects relevant for the task into fewer groups (Experiment 1) than did those in high likelihood conditions. In other words, participants who thought it was unlikely that they were going to perform the future tasks represented them in more abstract terms.

### True and False Intentions

To discriminate between true and false intentions is a specific form of deception detection that focuses on future events. For half a century deception detection studies have been focusing on past events, and the first study on intentions was published only 6 years ago (Vrij et al., 2011). This is surprising given that such knowledge has the potential to help legal professionals prevent future crimes (Granhag and Mac Giolla, 2014). For example, it could help investigators detect that a person is lying about planning a terrorist attack. In brief, a true intention refers to a future action which a person claims that s/he will perform, and which s/he also genuinely *intends* to perform. By contrast, a false intention refers to a future action which a person claims that s/he will perform, but which s/he actually *does not* intend to perform. In other words, a true intention refers to the mental representation of an alleged and genuinely intended future action, whereas a false intention refers to the mental representation of an alleged action not intended to be performed. Previous intention studies focused primarily on verbal cues to deceit (for a review see Granhag and Mac Giolla, 2014). This work uncovered some differences between truth tellers’ and liars’ statements of intent (e.g., Mac Giolla et al., 2013), as well as possibilities to further magnify these differences using active detection strategies such as interviewing techniques developed to elicit cues to deceit (Sooniste et al., 2014). For example, Warmelink et al. (2013) found that true statements of intent consisted of more detail than false statements of intent, and Mac Giolla et al. (2013) found that statements of true intentions referred more to ‘how’ the action were to be followed through, whereas statements of false intentions referred more to ‘why’ the action was to be performed. Despite these promising results, the literature is rather meager when it comes to theoretical understanding of true and false intentions. Previous studies have been loosely anchored in related fields such as research on planning (Sooniste et al., 2013), mental imagery (Knieps et al., 2013), and implementation intentions (Mac Giolla et al., 2013). Therefore, the current study takes a step back from mainly looking at verbal statements, to instead focusing on the mental representations of the future tasks which such statements of intent should be produced from. Specifically, we address the question whether people’s mental representations of true and false intentions differ systematically, and whether such differences can pave the way for novel cues to truth telling and deceit.

### New Directions: Applying CLT to True and False Intentions

Since intentions refer to future situations, not currently experienced, they must be represented by mental construals. Furthermore, considering the theoretical framework of CLT, there are reasons to believe that true and false intentions are represented at different levels of construal. This is because the hypotheticality, or the likelihood of the future event occurring, differs between true and false intentions. A true intention, since it comes with a commitment to perform the action in question, has a high likelihood of occurring (Malle and Knobe, 2001). A false intention, on the other hand, is not intended to be performed, and therefore has a low likelihood of occurring. In other words, in the context of true and false intentions, veracity is by definition confounded with the likelihood of the future events occurring. Based on research findings showing that likelihood influences construal level, we expect that having a true or false intention should likewise influence construal level. Specifically, true intentions should be more concretely construed, while false intentions should be more abstractly construed. Previous research findings indicate that true and false intentions may be construed at different levels. For example, Warmelink et al. (2013) found that truth tellers’ statements consisted of more detail than liars’ accounts, which could be due to differences between truth tellers’ and liars’ mental construals. Also, Knieps et al. (2013) found that truth tellers, in comparision to liars, were more likely to experience Episodic Future Thoughts (i.e., mental images), and to have more vivid EFTs related to their stated intentions. This is in line with CLT findings showing that pictures are related to lower-level, concrete, representations, while words are associated with higher-level, abstract, representations (Rim et al., 2014). Although the link between these studies and construal level is speculative, the findings are consistent with the predictions of CLT. Despite these indications, there have been no studies explicitly testing whether the mental construals of true and false intentions differ systematically. In fact, to our knowledge, no studies have used Construal Level Theory as an explanatory framework for how lies and truths are mentally represented.

### The Current Study

In contrast to previous studies on intentions, the current study examines and experimentally tests how truths and lies about the future are mentally represented. In two experiments we tested the relation between truth status (true vs. false intention) and construal level, measured with an online video segmentation task (Experiment 1) and participants’ preferences for either abstract or concrete descriptions of tasks (Experiment 2). We assessed how participants, who were asked to either lie or tell the truth about performing simple future tasks, mentally construed the future tasks. We predicted that false intentions would be represented more abstractly than true intentions, since false intentions refer to events that are less likely to occur.

## Experiment 1

In this experiment, we examined to what extent participants having a true intention differed from those having a false intention in how abstractly the task was construed. Participants watched a video clip of a simple task. Half were to truthfully communicate to another person that they would later perform the task (i.e., they communicated a true intention). Half were to deceptively communicate to another person that they would later perform the task (i.e., they communicated a false intention). While watching the clip, they were asked to divide what happened in the video (i.e., the task) into meaningful action units. This type of segmentation task has been used in previous CLT studies as a technique for measuring construal level (e.g., Henderson et al., 2006; Wakslak et al., 2006). In brief, a higher number of segments is indicative of a lower-level construal while a lower number is indicative of a higher-level construal.

We expected that liars would segment the behavior into fewer units than truth tellers, indicating a relatively higher-level of construal. We also included a control group. The participants in this group had a genuine intention to perform the task, and were in that regard similar to the truth telling group. However, they did not communicate their intention to another person (as did truth tellers). The control condition was included in order to examine if, and how, the communication of a statement plays a role in construing an intention.

### Method

#### Participants and Design

An *a priori* power analysis was conducted in order to estimate the number of participants needed to reach sufficient power of the experiment. Based on a medium-sized effect of psychological distance on construal level found in a recent meta-analysis (Soderberg et al., 2014), a sample size of 159 participants was needed to reach 80% power. Data collection was, however, stopped early. This was due to the results of Bayesian analyses indicating that adding more participants would unlikely have altered the results (see Results section). To sample data until the Bayes factor sufficiently favors one model over the other has been proposed as an acceptable decision-rule for optional stopping (as opposed to cases of Null Hypothesis Significance Testing; Rouder, 2014). It also provides the opportunity to efficiently spend available resources by not collecting more data than what is needed (Lakens, 2014).

One hundred and twenty-five participants took part in the experiment. Ages ranged from 19 to 68 (*M* = 28.47, *SD* = 8.86), and there were 88 females, 36 males, and one participant who did not specify their gender. Participants were recruited via a pool of voluntary participants at the Department of Psychology of a large Swedish University, which consists of both students and people from the general population. The study was described as a study of perception and communication.

Participants were evenly divided into one of three groups: truth tellers, liars, or control condition. All participants received 50 SEK (approximately 6 United States dollars) for participating in the study. In order to increase truth tellers’ and liars’ motivation, they were also promised an extra 50 SEK if they convinced another person that they told the truth. In reality, all participants in both of these groups received the extra compensation. Participants in the control group, since not uttering any statement, were instead included in a lottery in which one participant received two movie tickets as extra compensation.

#### Procedure

Random assignment to condition was determined in advance. However, slight deviations were made in certain cases (e.g., when there was no time for the experiment leader to switch from one condition to another to prepare material).

When arriving to the lab, participants signed an informed consent and were seated in front of a computer.

##### Instructions to truth tellers and liars

Truth tellers and liars were told that the study was about people’s ability to detect deceit. They were told that their task was to provide a statement regarding a future task for someone else to judge, and that this task was to build a simple cardboard toy car. Participants were told they would be randomly divided into either a truth telling group or a lying group of subjects, so in other words the participants were aware of the manipulation of veracity. Truth tellers were told they *were* going to build the toy car at the end of the experiment, and that they were first going to convince another person that they were indeed going to build the car (i.e., state a true intention). Liars were told they *were not* going to build this toy car, but to convince the other person that they were (i.e., state a false intention).

After reading these instructions, but before watching the video clip, participants were asked by the experiment leader to briefly summarize what was going to happen. All participants had correctly understood the instructions. In addition, truth tellers were also shown the material to be used for building the car, to further increase their belief that they were going to perform this task.

##### Instructions to control group

Participants in the control condition were not asked to communicate their intention to anyone. Instead, they were simply told that they were going to watch a video clip of how to build a toy car, and that they were to build the car themselves at the end of the experiment. Hence, they had a genuine intention to perform the task, but did not utter any statement regarding their intention. Like truth tellers, they were also shown the material to be used for building the car.

##### Video segmentation task

The video clip was 5 min long and depicted the hands of a person building the toy car (e.g., cutting a piece of cardboard, placing wheels on the car). Before watching the video clip, all participants received the following instruction, adapted from previous behavior unitization studies (e.g., Newtson, 1973) and Construal Level Theory experiments (e.g., Wakslak et al., 2006, Experiment 4):

We are interested in what units people use to organize and segment a behavior. What we mean by this is that different people use different ways to segment a behavior. To give an example: imagine a person who stands up, turns around, walks over the floor, closes a door, turns around, walks back, sits down. You could see each of these steps as separate and meaningful units. Or you could see it as three units: stands up, closes the door, sits down. Or you could see it as one meaningful unit: closes the door.

What we want you to do is to segment what happens in the video clip into what you consider to be natural and meaningful action units. Press the space bar when, according to you, one meaningful action unit ends and another begins. These should be whatever feels as natural and meaningful units to you. There is no right or wrong way of doing this. We just want to know how you do this.

After reading the instructions, participants watched the video clip and the number of clicks on the space bar were recorded.

##### Statement of intent

After the video segmentation task, truth tellers and liars provided their written statements of intent on the computer. They were led to believe that a person, seated in the next room, was going to judge whether the participant was lying or telling the truth. In reality, there was no other person judging their statements. Participants were given 5 min to complete their statement.

After having written their statements (for truth tellers), or directly after watching the video clip (for control condition), participants were intercepted and told that they were not going to build the toy car. Instead they were informed that they were going to answer some questions as a final part of the experiment. Liars were informed that they were no longer required to lie and that they were to answers the questions in a truthful manner.

##### Manipulation check measures and background variables

As manipulation-checks, participants were asked if they lied or told the truth (binary choice) as well as to rate on a 7-point Likert scale the extent to which they believed they were actually going to build a toy car (1 = *to a very low extent*, 7 = *to a very high extent*). Also, since it is well documented that people tend to process information more globally when in a positive mood, when being inattentive, as well as when perceiving a task as easy (Förster and Dannenberg, 2010), background variables concerning these aspects were collected. Ratings on participants’ mood and attentiveness were collected via the Positive and Negative Affect Scale (PANAS; Watson et al., 1988). Participants were also asked to what extent they focused on memorizing what they saw while watching the video clip (1 = *very low extent*, 7 = *very high extent*) and how difficult they perceived it to be to build the toy car (1 = *very easy*, 7 = *very difficult*).

### Results and Discussion

In total, four participants were excluded from analyses; three participants misunderstood the study instructions and one participant was excluded due to technical problems. This resulted in a final number of 40 participants in the truth telling condition, 39 participants in the lying condition, and 42 participants in the control condition. With a final sample size of 121 participants, the experiment was slightly underpowered at an estimated power of 68% (Hedges’ *g* = 0.475, alpha level set at 0.05; based on the effect size estimate of Soderberg et al., 2014).

#### Manipulation Checks and Preliminary Analyses

All truth tellers reported that they told the truth and all liars reported that they lied. A one-way Analysis of Variance (ANOVA) showed that groups differed in terms of the extent to which they believed they were going to perform the task, *F*(2,118) = 56.08, *p* < 0.001. *Post hoc* analyses (Tukey HSD) revealed that both truth tellers (*M* = 5.93, *SD* = 1.67) and participants in the control condition (*M* = 6.52, *SD* = 1.11) believed to a greater extent than liars (*M* = 2.87, *SD* = 2.07) that they were actually going to perform the task (both *p*s < 0.001). This indicates that true intentions were successfully created.

Three one-way ANOVAS were also conducted on factors previously known to correlate with level of information processing. First, there was a main effect on mood, *F*(2,118) = 5.68, *p* = 0.004. *Post hoc* analyses (Tukey HSD) showed that liars were in a less positive mood (*M* = 3.63, *SD* = 0.37) than both truth tellers (*M* = 3.87, *SD* = 0.33) and participants in the control condition (*M* = 3.83, *SD* = 0.28), both *p*s < 0.024. Second, there was a main effect on memorization, *F*(2,118) = 8.88, *p* < 0.001. *Post hoc* analyses revealed that liars focused on memorizing to a lesser extent (*M* = 4.90, *SD* = 1.86) than both truth tellers (*M* = 6.23, *SD* = 0.92) and participants in the control condition (*M* = 5.79, *SD* = 1.35) (both *p*s < 0.017). Finally, with a mean difficulty rating of 1.82 (*SD* = 1.11), participants rated the task as easy. The groups did not differ in terms of how difficult they perceived the task to be, *F*(2,118) = 1.71, *p* = 0.184.

#### Main Analysis

A one-way ANOVA was conducted to examine whether having a true or false intention influenced level of construal, measured by number of video segments. Results showed no difference in number of segments between participants having a true intention (*M* = 12.92, *SD* = 9.77), participants having a false intention (*M* = 12.54, *SD* = 9.11), and participants in the control group (*M* = 11.83, *SD* = 10.22), *F*(2,118) = 0.13, *p* = 0.875, η^2^ = 0.002, 95% CI [0.001, 0.015]. The critical effect size between the true and false intention conditions was *d*_s_ = 0.040, 95% CI [-0.408, 0.488]^1^. We also conducted a Bayesian Analysis of Variance (ANOVA) in order to examine the support for the null hypothesis. The analysis revealed a Bayes factor of 9.889 in favor of the null, indicating moderate-to-strong support for the lack of an effect^2^. This result is not in line with the main hypothesis and suggests that truth tellers and liars represented their intentions at similar, instead of different, levels of construal.

Mood and degree of memorization could theoretically influence one’s construal level of an event. Since these measures differed between experimental conditions, we reanalyzed the data in two separate ANCOVAs with memorization and mood as covariates. As in the main analysis, results revealed that construal level still did not differ significantly between groups when controlling for mood and memorization, *F*s < 0.17, *p*s > 0.845.

The results indicate that true and false intentions are construed at similar levels of abstraction. However, there are reasons to look closer at certain aspects of the study in order to gain insights to other potential sources of these null results. First, the null results may be due to the fact that construal level was only measured with one type of dependent variable (i.e., an online behavior segmentation task). In other words, although the segmentation task has been used previously to capture differences in how task related information is construed (Wakslak et al., 2006, Experiment 4), it does not allow one to generalize to the construal of information at a conceptual level. Second, substantial individual differences in the dependent variable (as evidenced by the large *SD*s) could be an explanation for the null findings; although similarly large SDs were reported by Wakslak et al. (2006, Experiment 4), who still found a large effect size on the same outcome measure (Cohen’s *d* = 1.54). Third, potential confounds, such as memorization and mood, differed between the groups. Based on the above, we used a repeated measures design in Experiment 2 in order to decrease individual and between groups variations. In addition, we introduced two other measures of construal level.

## Experiment 2

Using repeated measures in deception research has been proposed as a means of reducing individual variances (Watkins and Martire, 2015). We therefore chose to manipulate veracity within subjects in Experiment 2. Participants were introduced to a series of simple tasks by watching short video clips of them. They were asked to perform half of these tasks and not to perform the other half. In conjunction with each task, independently of whether they would perform it or not, they were asked to write down that they were going to perform it. In doing so, we elicited statements of true and false intentions from each participant. Drawing on the work of Wakslak et al. (2006, Experiment 3), these statements of intent, as well as participants’ preference for either abstract or concrete descriptions of the tasks, provided our two dependent measures of construal level. We predicted that participants would show a greater preference for abstract descriptions of the tasks in the cases of false intentions than true intentions, as this would be indicative of a higher level of construal.

### Method

#### Participants

Fifty-nine people participated in the experiment, and ages ranged from 18 to 79 (*M* = 28.36, *SD* = 12.54). Forty were female, 17 male, and two did not specify their gender. Participants were recruited from a pool of voluntary participants at the Department of Psychology of a large Swedish university, consisting of students and people from the general population. The study was described as a study about performing tasks. Each participant received 50 SEK (approximately 6 United States dollars) for participating in the study. Nine participants were excluded from analyses since they misunderstood the study instructions: three participants did not correctly follow the manipulation instructions, and six participants did not comply with constraints of statement length. Power analysis showed that with the final sample size of 50 participants, the experiment reached a power of 93% (based on Hedges’ *g* = 0.475, alpha level set at 0.05).

#### Design and Material

Veracity (true vs. false intention) was manipulated within subjects. Eight simple tasks were recorded on video, which could either be performed on the computer or by using props placed in the room where the experiment took place (see **Table 1**).

**Table 1:** Tasks and binary alternatives used in forced-choice dependent variable.

		Choice alternative	
Task	Description	Concrete	Abstract
Cabinet task	Locking a cabinet	Turn key	Secure object
Description task^a^	Describing a shape with text	Insert text box	Describe
Piano task^a^	Playing four notes on a keyboard	Press keys	Make music
Wiping task	Wiping a table with a cloth	Wipe with a cloth	Clean a table
Paper airplane task	Building a paper airplane	Fold paper	Make a toy
Lego task	Building a house with Lego	Put bricks together	Build a house
Paper bin task	Throwing paper in a bin	Crumple and throwing paper	Test one’s accuracy
Poster task	Attaching a poster on a wall	Attach paper	Decorate a wall

^a^*Tasks to be performed on the computer*.

In order to reduce the risk that participants would perceive the simple tasks as too mundane, we introduced an element of competition. A video camera recorded participants during the whole experiment. Participants were led to believe that the experiment leader would check so that all tasks were performed in line with the instructions. Participants were told that if they had done everything according to the instructions, they had the chance of winning two cinema tickets as extra compensation.

#### Procedure

Participants were seated in front of a computer and informed that they were going to perform a series of actions in the room they were in. They were told they would watch eight video clips depicting eight simple tasks. In addition, they were informed they would only perform half of these tasks (4), and not perform the other half (4). They were also told that after seeing each video clip, they would be asked to write down a description of the task. In the cases where they would actually *perform* a task, they would describe the task which they would actually perform (i.e., a true statement of intent). In the cases they would *not perform* a task they would fill in the box *as if* they were to perform it (i.e., a false statement of intent). Before starting the real experiment, participants had a practice round, matching a true intention round, in order to make them familiar with the procedure. The manipulation was given just before each video clip was shown, and was simply formulated as *You will/will not perform the task.*

After each clip, participants were asked which of two descriptions of the task that they thought best described the task. All tasks were given one more abstract descriptions and one more concrete description (see **Table 1**). Participants answers to these forced-choice questions were coded as either 1 (higher-level, abstract description) or 0 (lower-level, concrete description). Two construal level indexes were created for each participant; one for false intentions (ranging from 0 to 4) and one for true intentions (ranging from 0 to 4). Thus, a higher index indicated a greater preference for general, higher level, descriptions.

#### Data Coding

Participants’ descriptions of the tasks were coded by the first author who was blind to condition. Each description was coded into one of three categories: lower level than basic level (coded as -1), at basic level, (coded as 0), or higher level than basic level (coded as +1). The basic level was established through pilot-testing of the video material, in which other participants were asked to watch each of the clips and write down their spontaneous description of the task. We used the most commonly occurring description of each task as the basic level of that task. Two construal level indexes were created for participants’ descriptions; by averaging the codings, participants received an index between -1 and +1 for their true and false intentions, respectively.

### Results and Discussion

No order effects were found based on the order in which participants were introduced to the different tasks. A paired samples *t*-test on participants’ answers to the forced-choice questions showed no statistically significant difference in construal level between their true intentions (*M* = 1.68, *SD* = 1.17), and their false intentions (*M* = 1.80, *SD* = 1.11), *t*(49) = 0.71, *p* = 0.479, *d*_av_ = 0.105, 95% CI [-0.192, 0.402], BF_01_ = 5.107.^3^ This again indicates that true and false intentions were mentally represented at similar construal levels. A paired samples *t*-test on the codings of participants’ descriptions of the tasks showed no difference in construal level between their true intentions (*M* = -0.11, *SD* = 0.28) and false intentions (*M* = -0.12, *SD* = 0.26), *t*(49) = 0.17, *p* = 0.866, *d*_av_ = -0.037, 95% CI [-0.410, 0.332], BF_01_ = 6.412. In line with the findings in Experiment 1, the results speak against our hypothesis and instead indicate that true and false intentions, within the current experimental paradigm, are construed at similar levels of abstraction.

## Cross-Experimental Meta-Analysis

Since the combined results of two experiments provide a more reliable estimate of the true effect than a single experiment, we conducted a meta-analysis of the two experiments in order to better estimate the overall effect of veracity (true vs. false intention) on construal level. Each experiment represented a unit of analysis. For Experiment 1 we used the between-groups data from the true and false intention conditions (in which the dependent measure was number of segments). For Experiment 2 we used the weighted average of the two within-subjects dependent measures (preference for abstract/concrete description and codings of participants own descriptions). The Comprehensive Meta-Analysis software (Borenstein et al., 2006) was used to conduct the analysis. Using a random effects-model, an overall effect size of Hedges’ *g* = 0.02 was found (positive values indicating an effect in the predicted direction), 95% CI [-0.164, 0.214]. This result lends further support to the idea that true and false intentions may be represented at similar levels of mental construal in the current experimental paradigm. Our confidence in the null effect is furthered by the narrow confidence intervals. To put this result in context, the upper limit of the 95% confidence interval equals what is typically considered a small effect (Cohen, 1988), and is less than half the size of the average effect size (Hedges’ *g* = 0.475) of the most extensive meta-analysis to examine the effect of psychological distance on construal level (Soderberg et al., 2014).

In addition to the analysis above, we conducted a test for *equivalence*. Equivalence testing allows one to reject effects that fall outside a range of pre-specified equivalence bounds. It has been proposed as a way of testing if an observed meta-analytic effect is practically equivalent to zero, by falling within these bounds (Lakens, 2017). Using a narrow equivalence bound (Hedges’ *g* = -0.2 and 0.2), the equivalence test was significant, *p* = 0.035, 90% CI [-0.134; 0.184].^4^ This further supports the notion that there is no meaningful effect in our data.

## General Discussion

We proposed and tested the idea that since true (vs. false) intentions correspond to high (vs. low) likelihood events, they should be represented at a relatively lower (vs. higher) level of mental construal. This novel theoretical modeling rests on the previously established relation between likelihood and abstraction found within the CLT framework (Wakslak et al., 2006). Failing to support the predictions, the results indicate that true and false intentions are mentally represented at similar levels of abstraction. Results from Experiment 1 showed that truth tellers and liars did not differ with regards to the level at which they construed their intended or unintended task, measured by the number of video segments they divided the task into. The results from Experiment 2 showed that manipulating true and false intention within subjects did not alter their level of representation of a series of tasks. Instead, participants’ preferences for abstract or concrete descriptions of the tasks were unaffected by this manipulation. The very small effect size and narrow confidence interval around this estimate found in our cross-experimental meta-analysis further supports this notion. It is meanwhile worth underlining the need to be cautious when interpreting null results, since the lack of an effect may be due to many factors. We discuss the results below from the perspective of CLT as well as recent deception detection research.

### Implications for Construal Level Theory

The current results are at odds with previous studies on hypotheticality and construal level. A recent meta-analysis showed a medium-sized effect of likelihood on construal level (Soderberg et al., 2014). Of particular interest are the results of Wakslak et al. (2006) who found that likelihood influenced participants’ construal of *future* events. In six experiments they used different dependent measures, future events, as well as manipulations of likelihood. Their thorough investigation should be seen as strong support for the proposed effect of hypotheticality on construal level. Since the current study failed to replicate previous findings, it adds to cumulative research on the topic. Meanwhile, it raises the question why there was no effect of likelihood (which was manipulated through veracity) on construal level in the present study.

One factor that may account for the null findings are the sort of tasks introduced to participants. The current experiments and those of Wakslak et al. (2006, Experiment 4) examined relatively simple tasks. However, the tasks by Wakslak et al. (2006) were more unfamiliar (e.g., cutting and folding paper in different geometrical shapes) than those used in the current study (e.g., folding a paper airplane or attaching a poster to a wall). Past research has found that task difficulty and unfamiliarity correlates with the level of action identification. Specifically, difficult and unfamiliar tasks are generally identified at more concrete levels of abstraction compared to more familiar tasks (Vallacher and Wegner, 1989). Put differently, very familiar tasks are represented schematically, and do not require a focus on concrete steps in order to be performed. The tasks in the current experiments may have been too simple and familiar to allow for anything else than a high level, schematic representation. Also, the fact that the tasks were presented to participants via video clips may further have increased participants’ familiarity with the tasks. Hence, the choice of tasks and presentation medium may have led to more abstract schematic representations for both those stating a true and false intention, thereby canceling out any potential influences of hypotheticality. Future studies examining the effects of veracity on mental construal should therefore examine more complex or unfamiliar tasks.

A second potential explanation for the results is that hypotheticality status (high or low likelihood) may not equate to truth status (telling the truth or lying). The predicted relationship between construal level and truth status relies on this assumption. If it is not valid, we should not expect to see this pattern. Deception is characterized by, for example, its social function, which is something that hypotheticality does not necessarily entail. For example, deception can aid people in presenting themselves in a more positive light or be used as a social lubricant (Vrij, 2008). Trying to convince someone else that one is telling the truth (Experiment 1), or expressing deceptive as well as truthful statements (Experiment 2), may put participants in a different mindset compared to simply being led to think there is a high or low likelihood to perform a certain future task. In other words, since deception is a form of communication with specific social motives, it may represent a special case of mental construal. By mapping people’s mental representations of lies, future research can shed light on this issue and set the boundary conditions for what types of events and actions that can be accounted for within the CLT framework.

### Implications for True and False Intentions

Although the predicted relationship between true and false intentions and construal level did not receive empirical support, the current study contributes in several ways to the study of true and false intentions. Firstly, the current data suggest that when lying about a simple future task, the mental construal of that task may be similarly construed as when telling the truth. However, as argued in the section above, there are theoretical grounds for expecting differences between liars and truth tellers when tasks are more complex or unfamiliar. Though speculative, such a finding could have important practical implications. Let us return to the threat assessment example given in the section “Introduction.” Following the logic above, the nature of the action in question may be a crucial criterion for whether or not differences in statement abstraction are to be expected. If simple future tasks are similarly construed independently of veracity, one should not expect any differences in downstream effects (e.g., in level of ‘segmentation’ of statement) between liars’ and truth tellers’ statements. However, if the task is more difficult, this could result in differences in construal level, which could have different downstream effects on liars’, compared to truth tellers’, statements of intent.

Secondly, the current results contribute to the growing body of research on true and false intentions. Previous studies have shown verbal differences between true and false *statements* of intent, for example when it comes to level of detail. The fact that no differences emerged between liars and truth tellers’ *mental representations* of intent in the current context indicate that previously found verbal differences may be due to other factors than differences in mental construal. For example, differences between liars’ and truth tellers’ strategies to appear as honest (for a review, see Granhag et al., 2015) may have been a crucial cause of these verbal differences. However, mental representations of intentions should be further investigated in order to illuminate why verbal differences may or may not occur.

## Conclusion

The current study presents, to our knowledge, the first attempt to apply Construal Level Theory in a deception detection context. Inconsistent with our predictions, it seems that truths and lies about intended future behavior are represented on a similar level of abstraction, at least when tasks are relatively simple. The fact that truths and lies are similarly represented can further explain the difficulties involved in the elusive task of deception detection. Future studies should examine whether this trend holds even for more complex statements of true and false intent.

## Ethics Statement

According to the Regional Ethical Board in Gothenburg, Sweden, a full ethical review was not required for this experimental set-up. The research was carried out in accordance with guidelines of the Swedish Research Council. Participants in the study granted their written and informed consent.

## Author Contributions

SC has the main responsibility for data collection and statistical analyses. SC, EMG, PAG, and KA have shared responsibility for the remaining aspects of this research.

## Conflict of Interest Statement

The authors declare that the research was conducted in the absence of any commercial or financial relationships that could be construed as a potential conflict of interest.

## Footnotes

1 Six extreme outliers (values larger than 1.5 interquartile ranges over the 3^rd^ quartile) were identified, and therefore a second one-way ANOVA, excluding these data points, was conducted. However, excluding these data points did not alter the outcome of this test, *F*(2,112) = 0.94, *p* = 0.395, η^2^ = 0.016, 95% CI [0.001, 0.062].

2 A default Bayesian approach was used with Cauchys prior width set at 0.707.

3 Due to the high attrition rate (15%), two separate *t*-tests, including all participants, were conducted on both dependent measures. Including these participants did not alter the outcomes of the tests; there was neither a statistically significant difference between participants’ answers to the forced-choice question, *t*(58), *p* = 0.446, *d*_av_ = 0.121, 95% CI [-0.187, 0.430], BF_01_ = 5.306, nor a statistically significant difference between the codings of participants’ own descriptions of the tasks, *t*(58) *p* = 0.896, *d*_av_ = 0.037, 95% CI [-0.267, 0.341], BF_01_ = 6.966.

4 The package ‘TOSTER’ for R was used for conducting this analysis (Lakens, 2017).
